# Percutaneous fractionated radiotherapy of the groin to eliminate lymphatic fistulas after vascular surgery

**DOI:** 10.1186/s40001-023-01033-6

**Published:** 2023-02-09

**Authors:** Danny Jazmati, Bálint Tamaskovics, Norman-Philipp Hoff, Bernhard Homey, Edwin Bölke, Belebenie Boyomo, Waseem Garabet, Jan Haussmann, Wilfried Budach, Judith Neuwahl, Hubert Schelzig, Stefanie Corradini, Martijn van Griensven, Johannes Fischer, Wolfram Trudo Knoefel, John Pegani, Alessia Pedoto, Gerald Antoch, Julian Kirchner, Tom Lüdde, Noemi F. Freise, Torsten Feldt, Björn-Erik Ole Jensen, Verena Keitel, Christiane Matuschek

**Affiliations:** 1 Department of Radiation Oncology, University Hospital Dusseldorf, Medical Faculty, Heinrich-Heine University, Moorenstr. 5, 40225 Düsseldorf, Germany; 2 Department of Dermatology, Medical Faculty, Heinrich Heine University Hospital Dusseldorf, Düsseldorf, Germany; 3 Department of Vascular Surgery, Medical Faculty, Heinrich Heine University Hospital Dusseldorf, Düsseldorf, Germany; 4 Department of Radiation Oncology, LMU University of Munich, Munich, Germany; 5 MERLN Institute for Technology-Inspired Regenerative Medicine, Department cBITE, Maastricht University, Maastricht, The Netherlands; 6 Institute for Transplant Diagnostics and Cell Therapeutics, University Hospital, Leipzig, Germany; 7 Department of Surgery and Interdisciplinary Surgical Intensive Care Unit, University Hospital Dusseldorf, Medical Faculty, Heinrich-Heine-University, Dusseldorf, Germany; 8 Antom-E Systems, Houston, TX USA; 9 Department of Anesthesiology and Critical Care Medicine, Memorial Sloan Kettering Cancer Center, New York, NY USA; 10 Department of Diagnostic and Interventional Radiology, Medical Faculty University Hospital of Dusseldorf, Dusseldorf, Germany; 11 Department of Gastroenterology, Hepatology and Infectious Diseases, University Hospital Dusseldorf, Medical Faculty, Heinrich-Heine-University, Dusseldorf, Germany; 12 Department of Gastroenterology, Hepatology and Infectious Diseases, University Hospital Magdeburg, Medical Faculty, Otto-Von-Guericke-Universität Magdeburg, Magdeburg, Germany

**Keywords:** Surgery, Wound healing, Radiation therapy, Side effects, Benign disease, Amputation

## Abstract

**Background:**

Vascular surgery of the inguinal area can be complicated by persistent lymphatic fistulas. Rapid and effective treatment is essential to prevent infection, sepsis, bleeding, and possible leg amputation. Current data on irradiation of lymphatic fistulas lack recommendation on the appropriate individual and total dose, the time of irradiation, and the target volume. Presumably, a dose of 0.3–0.5 to 1–12 Gy should be sufficient for the purpose. Currently, radiotherapy is a “*can*” recommendation, with a level 4 low evidence and a grade C recommendation, according to the DEGRO S2 guidelines. As part of a pilot study, we analyzed the impact and limitations of low-dose radiation therapy in the treatment of inguinal lymphatic fistulas.

**Patients and methods:**

As a part of an internal quality control project, patients with lymphatic fistulas irradiated in the groin area after vascular surgery for arterial occlusive disease (AOD) III-IV, repair of pseudo aneurysm or lymph node dissection due to melanoma were selected, and an exploratory analysis on retrospectively collected data performed.

**Results:**

Twelve patients (10 males and 2 females) aged 62.83 ± 12.14 years underwent open vascular reconstruction for stage II (*n* = 2), III (*n* = 1), and IV (*n* = 7) arterial occlusive disease (AOD), lymph node dissection for melanoma (*n* = 1) or repair of a pseudoaneurysm (*n* = 1). Surgical vascular access was obtained through the groin and was associated with a persistent lymphatic fistula, secreting more than 50 ml/day. Patients were irradiated five times a week up to a maximum of 10 fractions for the duration of the radiation period. Fraction of 0.4 Gy was applied in the first 7 cases, while 5 patients were treated with a de-escalating dose of 0.3 Gy. There was a resolution of the lymphatic fistula in every patient without higher grade complications.

**Conclusion:**

Low-dose irradiation of the groin is a treatment option for persistent lymphatic fistula after inguinal vascular surgery.

## Introduction

Lymphatic fistula is defined as a connection between a lymphatic vessel and the skin, resulting in a persistent leakage [1, 2]. It occurs in 5–10% of vascular procedures in the groin area [1, 3–7], due to the close anatomical relationship between lymph nodes and vessels [8].

Technically challenging lymph node dissection can lead to persistent injury of the lymphatic vessels, resulting in a lymphatic fistula, especially in case of revisions and reoperations. Persistent lymphatic leak can promote bacterial contamination of the wound, leading to bacteraemia and sepsis [8]. Bleeding and haemorrhagic shock have been reported after these cases [8]. Hypoperfusion of the area can affect wound healing, with peripheral necrosis and the potential for limb amputation.

Lymphatic fistula is usually diagnosed on postoperative day 7, in the presence of lymphatic drainage above 50 millilitres per day. Management includes either a conservative or surgical approach [2]. Most cases resolve with the former, leaving surgery for infected cases, very large amounts of lymphatic drainage, or when conservative treatment fails. Surgical interventions include peripheral lymphatic ligation and vascularized muscle flaps [5].

Bed rest with negative pressure dressing and doxycycline administration are the conservative treatment of choice [2, 3]. Radiotherapy has been reported in the literature as a promising additional treatment [4, 5, 9, 10]. Low-dose irradiation of the groin is a very effective and safe option, it is well tolerated by the patient [7, 11] and it has a reported success rate between 67 and 100%. However, as data are scarce, optimal fractionation, dose, timing, and target volume definition remain uncertain.

In this pilot study, we describe our institutional experience of treating lymphatic fistulas after vascular surgery in the inguinal area with low-dose irradiation.

## Patients and methods

### Patient selection

This retrospective study identified patients who underwent radiotherapy in the University Hospital Düsseldorf for a lymphatic inguinal fistula between 01/2010 and 06/2022. The Ethical Board of the Heinrich-Heine-University Düsseldorf approved the study (#2022–2079), which was conducted in accordance with the Declaration of Helsinki and its later amendments. The decision for radiotherapy was determined by a multidisciplinary board composed of representatives from surgery, radiotherapy, and radiology. The target goal was to provide prompt radiotherapy within 72 h after diagnostic confirmation of lymphatic fistula.

### Treatment

Each patient underwent a simulation CT scan with a 3 mm slice thickness for planning the applied radiation dose. The clinical target volume included the drainage channel and the surrounding CT-morphologically visible tissue alterations with a 2 cm safety margin, which was anatomically adapted. The planning target volume (PTV) had a 6–7 mm safety margin on the clinical target volume (CTV).

Irradiation was planned to a total dose of 3–4 Gy using a single dose of 0.3 or 0.4 Gy, via IMAT (Intensity Modulated Arc Therapy) and MV (MegaVolt) photons.

The fistula was clinically monitored daily. The irradiation was terminated once the lymphatic drainage subsided.

### Follow-up

As part of our routine protocol, each patient underwent regular interdisciplinary follow-up appointments. This included a medical examination of the fistula, an assessment of radiotherapy-related adverse effects and checks for wound healing. Similarly to oncological radiotherapy treatments, side-effects were classified according to Common Terminology Criteria for Adverse Events (CTCAE) version 4.

Patients who were unable to attend their scheduled follow-up appointments were assessed via telemedicine.

## Results

Twelve patients, 10 males and 2 females with a mean age of 62.83 ± 12.14 years were eligible for inclusion in this investigational study. Patients underwent surgery in the groin for stage II (*n* = 2), III (*n* = 1), or IV (*n* = 7) arterial occlusive disease (AOD), lymph node dissection secondary to melanoma (*n* = 1) and repair of a pseudo aneurysm (*n* = 1). Patients’ characteristics are shown in Table 1. They all developed a lymphatic fistula within 7 days from their original operation, with a median volume of secretion of 200 ml/day. On average, each patient was irradiated with 6 (range 2–10) fractions per day before successful closure.

**Table 1:** Patient characteristics, radiation therapy, outcome and toxicity

Nr	Age	Sex	Procedure	Comorbidity	Beam energy & planning method	Number of fractions	Fraction dose (Gy)	Total PTV volume (cm^3^)	Total RT dose (Gy)	Response	Treatment-related side effects
1	69	Female	Vascular bypss grafting	AOD stage IV HTN nicotine abuse	6 MV IMAT	8	0.3	393	2.4	No lymph fistula after RT	No
2	69	Male	Vascular bypss grafting	AOD stage IV,OPD alcohol abuse CAD depression	6 MV IMAT	5	0.3	417	1.5	No lymph fistula after RT	No
3	77	Male	Vascular bypss grafting	AOD stage IV NIDDM, HTN CAD	6 / 15 MV 3D-CRT	5	0.4	714	2	No lymph fistula after RT	No
4	80	Male	Vascular bypss grafting	AOD stage IIb on both sides 3-vessel CHD left ventricular failure hyperlipidemia hyperuricemia	6 MV IMAT	2	0.4	443	0.8	No lymph fistula after RT	No
5	63	Male	Vascular bypss grafting	AOD stage IIa left side status post STEMI of the front cardiac wall hypertension status post esophageal carcinoma nicotine abuse (70 PY) iron deficiency anemia	6 MV IMAT	10	0.3	683	3	No lymph fistula after RT	No
6	61	Male	Vascular bypss grafting	AOD stage III left heterozygous factor V Leiden mutation, BA Sicca, myopia	6 MV IMAT	2	0.4	627	0.8	No lymph fistula after RT	No
7	77	Male	Vascular bypss grafting	AOD stage IV left 3-vessel CHD NIDDM, obesity chronic bilateral venous insufficiency, Hach stage IV, AF	6 MV IMAT	8	0.3	567	2.4	No lymph fistula after RT	No
8	45	Male	Left inguinal lymph node dissection	melanoma, cT0cN0pM1a stadium IV AJCC autoimmune colitis grade III	6 MV IMAT	4	0.3	450	1.2	No lymph fistula after RT	No
9	43	Male	Vascular ligationy	pseudo aneurysm after prosthetic loop shunt for dialysis autosomal dominant polycystic kidney disease (ADPKD),, hemodialysis secondary hyperparathyroidism hepatitis C, hepatic steatosis	6 MV IMAT	10	0.4	576	4	No lymph fistula after RT but prolonged duration	No
10	58	Male	Vascular bypss grafting	AOD stage. IV right side chronic nicotine abuse, chronic Leriche syndrome, adrenal adenoma	6 / 15 MV 3D-CRT	3	0.4	426	1.2	No lymph fistula after RT	No
11	59	Male	Vascular bypss grafting	AOD stage IV left side balance and speech disorders status post apoplexy 3-vessel-CHD combined mitral and aortic valve regurgitation CRF status post Renal transplantation	6 / 15 MV 3D-CRT	4	0.4	647	1.6	No lymph fistula after RT	No
12	53	Female	Vascular bypss grafting	AOD stage IV right side	6 MV IMAT	3	0.4	1082	1.2	No lymph fistula after RT	No

A single dose of 0.4 Gy per day was used in 7 patients, while 5 patients received a single dose of 0.3 Gy per day, five times a week. The mean planned target volume of radiation corresponded to 585 cm^3^. Obliteration of all lymphatic vessels occurred after a median cumulative dose of 1.2 Gy.

The closure of the lymphatic fistula occurred in every patient within the irradiation series, on average after 5.1 days. Acute and late radiogenic side-effects were not detected in any of the patients.

**Fig. 1:**
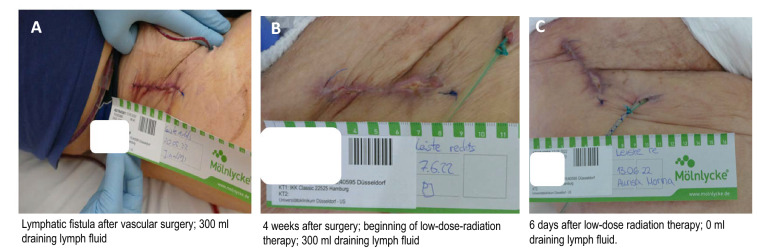
Patient with lymphatic fistula after vascular surgery. Panel **A** lymphatic fistula after 4 weeks of surgery, with more than 300 ml per day; Panel **B** wound at the beginning of radiotherapy; Panel **C** after 6 days of radiation therapy, the fistula is closed

A representative clinical picture of a patient after vascular surgery is shown in Fig. 1. A 59-year-old male developed a lymphatic fistula secreting more than 300 ml per day. Conservative therapy such as compression of the area did not improve the clinical scenario. Four weeks after surgery, radiation therapy with 10 × 0.3 Gy was initiated. After applying six fractions (cumulative 1.8 Gy), the leakage stopped and radiation therapy was terminated. The patient was discharged from the hospital after 3 days with an improved wound healing (Table 2).

**Table 2:** Fractionation schemes, therapeutic modalities, effectiveness and reported side effects of the radiation therapy of lymphatic fistulas

Cohort	Treatment period	Number of treated fistulas	Fraction dose	Number of fractions	Beam energy	Response rate	Treatment-related side effects
Middlesex (Croft 1978 [15])	1978	1	3.0 Gy	5	N.A	100%	N.A
Karlsruhe (Neu 2000 [4])	1989–1998	26	1.0 Gy	3–12	7–18 MeV e^−^	92%	N.A
Regensburg I (Dietl 2005 [5])	1997–2000	28	3.0 Gy	3–5	120–300 kV	96%	0
Graz (Mayer 2005 [10])	1987–2002	11 8	0.3–0.5 Gy 1.0–2.0 Gy	up to 16 up to 8	100 kV 4–11 MeV e^−^	82% 75%	0
Kazan (Kamalov 2010 [12])	2009–2010	5	0.06–0.3 Gy	3–10	180 kV	100%	N.A
Zaporozhye (Buga 2012 [14])	2008–2012	58	0.4–0.5 Gy	2–5	180 kV	67%	0
Regensburg II (Hautmann 2021 [7])	2005–2016	206	3.0 Gy	up to 6	6/15 MV	88%	0
Regensburg BHB (Uhl 2020 [1])	2007–2018	50	1.0 Gy	3–10	N.A	78%	0
Santander (Cañón 2021 [9])	2008–2018	43	1.5 Gy	3–5	6–18 MV	93%	0
Düsseldorf (present work)	2019–2022	12	0.3–0.4 Gy	2–10	6–15 MV	100%	0

**Fig. 2:**
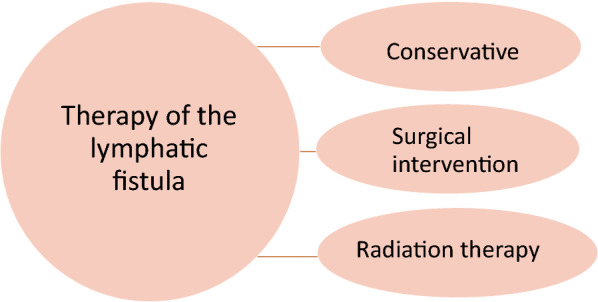
Therapeutic options for treating a lymphatic fistula. Our protocol is presented in the last row

**Fig. 3:**
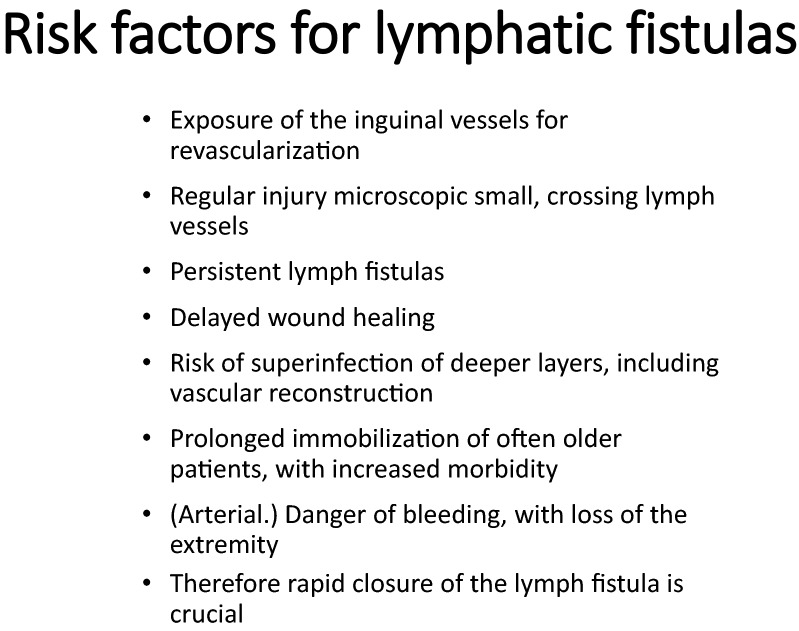
Possible complications of lymphatic fistulas

**Fig. 4:**
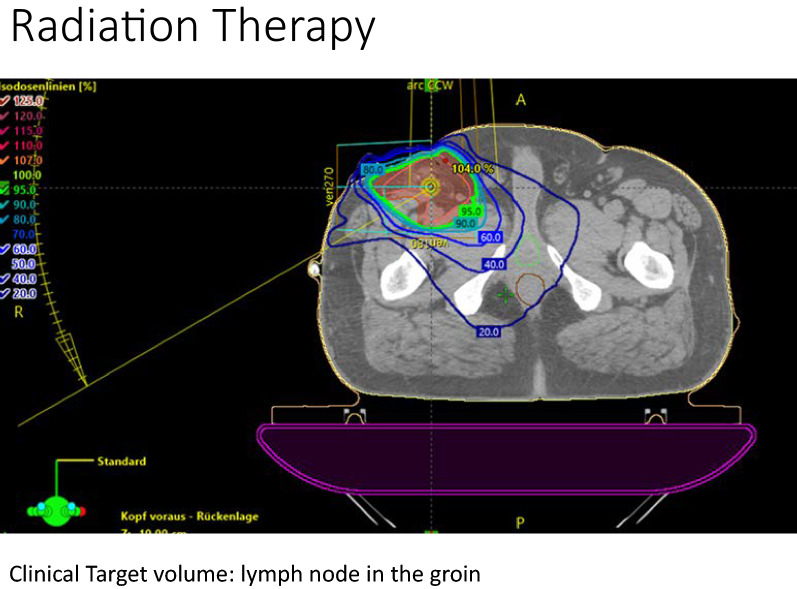
Dose distribution of an intensity modulated arc therapy plan in a representative axial section. The clinical target volume is defined as the drainage channel with 2 cm anatomically adapted margin and the superficial and deep inguinal lymph nodes and vessels in the operating field

**Fig. 5:**
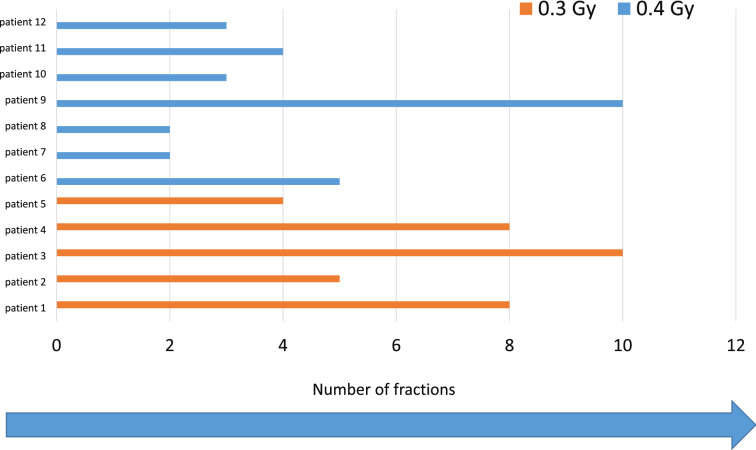
Number of fractions for each patient until the fistula is closed

The therapeutic options for treating a lymphatic fistula are presented in Fig. 2, while Fig. 3 presents the possible complications of lymphatic fistulas. The dose distribution of an intensity modulated arc therapy plan in a representative axial section is shown in Fig. 4. The clinical target volume is defined as the drainage channel with 2 cm anatomically adapted margin, with the superficial and deep inguinal lymph nodes and vessels in the operating field. The numbers of fractions each patient received until the closure of the fistula are presented in Fig. 5.

## Discussion

Lymphatic fistulas secondary to vascular surgery of the groin can be treated with conservative therapy, surgery, or radiation therapy. Persistent lymphatic fistulas can cause delayed wound healing, infection of deeper layers including the vascular graft, and a prolonged hospital stay. Immobility is associated with increased morbidity, wound infections and arterial bleeding, possibly leading to leg amputation.

Our study suggests a high efficacy and tolerability of low-dose irradiation for the treatment of lymphatic fistulas. This is in accordance with a trial from Mayer et al. from Graz [10]. They were the first to demonstrate lymphatic obliteration with low-dose radiation therapy. In their analysis, 13 of 17 patients had a complete response to low-dose radiation. There was a clinical response with total doses of ≤ 3 Gy and with fraction sizes ranging from 0.3 to 0.5 Gy [10]. Our results are consistent with this data. Mayer et al. used electrons or kV radiation therapy [4, 10] because that was standard of practice in the early 2000 and easy to apply. We used IMAT and MV photons with 6–15 MV which allowed to apply the smallest therapeutic dose to the target with minimal side effects to the surrounding tissue. In our cohort, successful obliteration of all the lymphatic vessels occurred after a median cumulative dose of 1.2 Gy, which is much lower of what reported in the literature. We think this is probably related to the direct application of the radiation to the clinical target volume of the lymph node area at the surgical site.

Different doses of radiation have been proposed to treat lymphatic fistulas [7, 8, 12, 13]. In the largest study to date, Hautmann et al. reported the use of 3 × 3 Gy at the start of the treatment [7] with an increase to 3 additional fractions of 3 Gy for persistent fistulas. The radiation treatment is usually stopped when the fistula output is less than 50 ml/ 24 h. In this cohort of 206 patients, 40.8% of individuals were irradiated with 9 Gy while 18 Gy were used in 46% of the individuals. In a large proportion of patients, a dose of 9 Gy in 3 Gy per fraction was not able to close the fistula. These doses are higher than what reported by Mayer et al. who suggested that a median dose of 2.4 Gy led to the fistula obliteration [7].

Our data are consistent with what reported by Graz, Kazan and Zaporozhy. Low fraction doses of 0.3–0.5 Gy do not seem to be inferior to higher doses [10, 12, 14]. The “As low as reasonably achievable” (ALARA) fundamental principle of radioprotection is very well applicable to this clinical use of radiation. It has been postulated that low-dose radiation leads to functional changes in the vessels, as well as decreased expression of E-selectin in the endothelial cells, decreased leukocyte adhesion, and reduced L-selectin expression.

Irradiating with electrons or low energy (kV) X-rays provides the benefit of deep tissue doses. If the clinical target volume incorporates deep inguinal lymphatic structures, the rotational intensity modulated high energy (MV) photon therapy could deliver the optimal dose coverage for the clinical target volume with the best healthy tissue protection. The research groups from Regensburg II, Santander and Düsseldorf worked with this technique.

The novelty of our treatment is the use of low-dose radiation at the site of the fistula in the immediate postoperative period. Hautmann et al. investigated the time sequence for starting radiation therapy after lymph node dissection (< 10 and more > 10 days), without finding a time correlation [15]. We believe that if radiation therapy is effective, the procedure should be performed as soon as possible to improve clinical outcomes, reduce costs, expedite discharge and improve the overall quality of life.

The radiation treatment of lymphatic fistulas has a very low carcinogenic risk. The lifetime risk of leukaemia is ≤ 0.2% [2], while the risk of developing basal cell carcinoma is approximately 0.006% [2]. On the other hand, people who undergo vascular surgery usually are elderly and with multiple co-morbidities, like coronary heart disease, dementia, and diabetes mellitus. Their life expectancy is reduced when compared to the younger counterpart, making the long-term radiogenic side effects negligible in this patient population.

Due to a large proportion of fistulas closing spontaneously, the benefits of prompt irradiation must be weighed against the risk of unnecessary treatment [4]. Prospective randomized trials are needed to answer this question.

## Conclusion

Low-dose radiation of the groin in the presence of persistent lymphatic fistula as a complication of vascular surgery in this area could be a useful therapeutic option to prevent wound infections possibly leading to limb amputation. Prospective trials are needed for determining the optimal dose of radiation and the target volume. Efficacy should be proven in a prospective randomised trial.

## Data Availability

All data and materials can be accessed via CM and DJ.

## Abbreviations

ALARA: As low as reasonably achievable
AOD: Arterial occlusive disease
CTCAE: Common terminology criteria for adverse events
CTV: Clinical target volume
DEGRO: Deutsche Gesellschaft für Radioonkologie
Gy: Gray
IMAT: Intensity Modulated Arc Therapy
KV: KiloVolt
MV: MegaVolt
PTV: Planning target volume
